# Structural and optical properties of position-retrievable low-density GaAs droplet epitaxial quantum dots for application to single photon sources with plasmonic optical coupling

**DOI:** 10.1186/s11671-015-0826-2

**Published:** 2015-03-10

**Authors:** Eun-Hye Lee, Jin-Dong Song, Il-Ki Han, Soo-Kyung Chang, Fabian Langer, Sven Höfling, Alfred Forchel, Martin Kamp, Jong-Su Kim

**Affiliations:** Center for Opto-Electronic Convergence Systems, Korea Institute of Science and Technology, Seoul, 136-791 South Korea; Institute of Physics and Applied Physics, Yonsei University, Seoul, 120-749 South Korea; Technische Physik, Universität Würzburg, Am Hubland, 97074 Würzburg, Germany; Department of Physics, Yeungnam University, Gyeongsangbuk-Do, 712-749 South Korea

**Keywords:** Quantum dot, Droplet epitaxy, Micro-photoluminescence, Single photon, GaAs

## Abstract

The position of a single GaAs quantum dot (QD), which is optically active, grown by low-density droplet epitaxy (DE) (approximately 4 QDs/μm^2^), was directly observed on the surface of a 45-nm-thick Al_0.3_Ga_0.7_As capping layer. The thin thickness of AlGaAs capping layer is useful for single photon sources with plasmonic optical coupling. A micro-photoluminescence for GaAs DE QDs has shown exciton/biexciton behavior in the range of 1.654 to 1.657 eV. The direct observation of positions of low-density GaAs DE QDs would be advantageous for mass fabrication of devices that use a single QD, such as single photon sources.

## Background

Low-density semiconductor quantum dots (QDs) have been one of the promising candidates in quantum information applications, such as single photon sources [1,2]. Less than 10 QDs per μm^2^ are preferred for a single photon source application, since optical interference from neighboring QDs could be avoided. Recent results of the growth of low-density QDs have shown that the size, density, and optical quality of QDs are sufficiently controllable [3-7]. In spite of advanced research on the application of low-density QDs, however, these QDs have been exploited in the scale of fundamental research. This is mainly attributed to the unknown position of an appropriate single QD capped for optical measurement. Optical measurement of low-density QDs was based on micro-photoluminescence (μ-PL). Most of the measurement time has been consumed finding the appropriate single QD, among the patterns fabricated for μ-PL measurement. Because there is no information on the QDs in those patterns, this selection process to find out the position of an appropriate single QD is controlled statistically, shows low yield, and as a result, becomes a bottleneck problem to overcome for a QD-based quantum information application.

Recently, in the case of InAs/GaAs QDs, many researchers have suggested a few solutions for this problem. Researchers have reported the result on positioning and pattering of single InAs QDs by two lasers (one for the positioning of appropriate single QDs by PL measurement, the other for developing photoresist) [8]. Cathodoluminescence can be a direct method to obtain the position of one single QD [9]. Regardless of their successful demonstration on the positioning of a single QD, laborious work with sensitive and expensive instrumentation remains necessary. Hennessy and Badolato et al. have shown two kinds of positioning techniques for InAs QDs in a photonic crystal nanocavity [10,11]. One of the methods is positioning by multi-stacked QDs, and another method is that by control of capping layer. In the case of latter one, the formation of the 1 ~ 2-nm hill on the position of a QD was observed by atomic force microscopy (AFM) [10]. However, the 1 ~ 2-nm hill may not be detected on scanning electron microscopy (SEM). Since SEM is widely used at a post-fabricating process with electron-beam lithography instruments, optimization for the capping condition on SEM would be needed in retrieving QDs.

The droplet epitaxy (DE) method has the merit of the growth of various quantum structures, such as quantum rings, disks, coupled QDs, and concentric quantum double rings, as well as low-density QDs, due to a perfect separation of groups 3 and 5 [12-15]. In addition, larger GaAs DE QDs can be grown on AlGaAs without strain effect, for larger oscillation strength, compared with InAs QDs on GaAs.

For low-density GaAs DE QDs, easier handling of them has huge merit in a silicon-based industry, since GaAs DE QDs as a single photon source with an emission of approximately 700 nm could be applied to low-cost and more sensitive silicon array detectors. Consequently, an efficient fabrication and measurement of quantum structures, based on DE growth, would be helpful for mass production of various devices.

In this letter, the authors will present the growth and position retrieval of low-density GaAs DE QDs of approximately 4 QDs/μm^2^. The positions of low-density GaAs DE QDs were directly observed on the surface of an Al_0.3_Ga_0.7_As capping layer by SEM, with optical signal from the DE QDs. High quality of optical properties with thin AlGaAs capping will be useful for the application to single photon sources with plasmonic optical coupling [9,16].

## Methods

**Figure 1:**
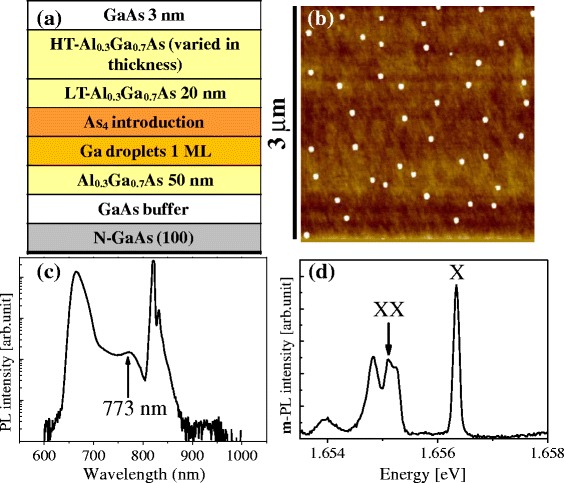
**Schematics of structures, AFM image, and the PL peaks of low-density GaAs DE QDs. (a)** Schematics of structures of low-density GaAs DE QDs as a function of thickness of HT-Al0.3Ga0.7As (2, 25, 50, 85 nm), **(b)** AFM image (3 μm × 3 μm) of low-density GaAs DE QDs (approximately 4 QDs/μm^2^) on Al0.3Ga0.7As/GaAs substrate, and **(c)** macro-PL (log scale) and **(d)** μ-PL (exciton, biexciton peaks) spectra of low-density GaAs DE QDs with 105-nm capping at low temperature (12 ~ 16 K).

GaAs DE QD samples were grown by a molecular beam epitaxy (MBE) system. Figure 1a shows a full sample structure, with low-density GaAs DE QDs (approximately 4 QDs/μm^2^) on an Al_0.3_Ga_0.7_As/GaAs substrate, with a variable thickness Al_0.3_Ga_0.7_As cap layer. After deoxidation at approximately 600°C under As_4_ and Al_0.3_Ga_0.7_As/GaAs buffer layer growth, formation of low-density Ga droplets was tested to predict the diameter, height, and density of GaAs DE QDs. Figure 1b indicates an AFM image of low-density GaAs DE QDs used in the full structure of Figure 1a. The As_4_ flux with beam equivalent flux of 1 × 10^−5^ Torr during 60 s was introduced on low-density Ga liquid droplets, as in Figure 1b, near room temperature. After As_4_ injection, a 20-nm-thick Al_0.3_Ga_0.7_As (LT-Al_0.3_Ga_0.7_As) layer covered low-density GaAs DE islands at near room temperature, to retain the shape of GaAs DE islands. Then, an additional Al_0.3_Ga_0.7_As layer (HT-Al_0.3_Ga_0.7_As) was grown, increasing the substrate temperature up to approximately 580°C. The thickness of the additional Al_0.3_Ga_0.7_As layer was varied and of 2, 25, 50, and 85 nm (the total thickness of Al_0.3_Ga_0.7_As was 22, 45, 70, 105 nm, respectively). The top of Al_0.3_Ga_0.7_As-capped samples was covered by 3-nm-thick GaAs. Rapid thermal annealing (RTA) was performed at 850°C for 240 s with ambient Ar, to improve crystallinity of GaAs DE islands. A macro-PL and μ-PL at low temperature (12 ~ 16 K) were measured by 532-nm laser to verify the formation of quantum structures, depending on the thickness of capping layers, and excitonic states of low-density GaAs DE QDs.

## Results and discussion

Figure 1b shows an AFM image of low-density GaAs DE QDs obtained by a change of growth conditions, such as growth temperature, Ga flux, and Ga coverage. Low-density GaAs DE QDs in Figure 1b showed 81.6 ± 9.5 nm in diameter, 14.7 ± 1.7 nm in height, and 4.4 QDs/μm^2^. Typically, in our case, after injection of As_4_, the change of GaAs DE QDs in density and diameter was negligible, compared with those of the Ga droplets. However, the height of the GaAs DE QDs was approximately two times larger than that of the Ga droplets. The height of Ga droplet is in the range of 7 ~ 8 nm. Here, the authors should point out that the shape of the GaAs DE QDs is not identical when they are grown on the surface and buried in the AlGaAs matrix. In the case of GaAs DE QDs of reference [7], the size of GaAs DE QDs on the surface was approximately 12-nm height and approximately 60-nm width and found that approximately 9-nm height and approximately 35-nm width when they were placed in the AlGaAs matrix. Furthermore, the effective size of GaAs DE is additionally reduced due to post-thermal annealing/intermixing and finally gives us the low-temperature PL peaks around 1.70 ~ 1.80 eV. Considering larger size of the GaAs DE QDs in this experiment as a starting size on the surface, the PL peaks in Figure 1c around 1.60 eV (approximately 0.773 nm) are attributed to the GaAs DE QDs after AlGaAs capping and post-annealing/intermixing.

A macro-PL of low-density GaAs DE QDs with 105-nm Al_0.3_Ga_0.7_As cap layer at 12 K was observed after RTA. Low-density GaAs DE QDs were formed by As_4_ injection on Ga droplets of Figure 1b. The emission of low-density GaAs DE QDs was verified at approximately 773 nm in Figure 1c. Based on the result of macro-PL, a μ-PL of low-density GaAs DE QDs (approximately 4 QDs/μm^2^) at 16 K was performed. Figure 1d shows exciton (X)/biexciton (XX) behavior in the range of 1.654 to 1.657 eV. Exciton and biexciton behavior can be characterized by the value of biexciton binding energy [17]. A few studies have reported 3 to 5 meV for the biexciton binding energy of GaAs QDs [17,18]. The diameter of QDs in such studies was less than approximately 50 nm. In our case, the biexciton binding energy was approximately 1.2 meV, and the diameter of QDs was 70 ~ 90 nm. The relatively small biexciton binding energy for the present QDs would be attributed to a large size of QDs, since an increase of radius of QDs reduces biexciton binding energy [19]. For the exciton peak, a full width at half maximum (FWHM) showed approximately 136 μeV. Mano et al. have reported that narrow linewidth around 35 μeV has been observed from an emission of a single GaAs DE QD [6]. Since the value of FWHM depends on the local environment, regardless of QD emission energy, this larger FWHM value could be reduced by improving the quality of the barrier in QD structure, for instance, by annealing during growth [6,20]. The optical properties of low-density GaAs DE QDs of the authors’ sample are covered in detail at the other articles of reference [21].

**Figure 2:**
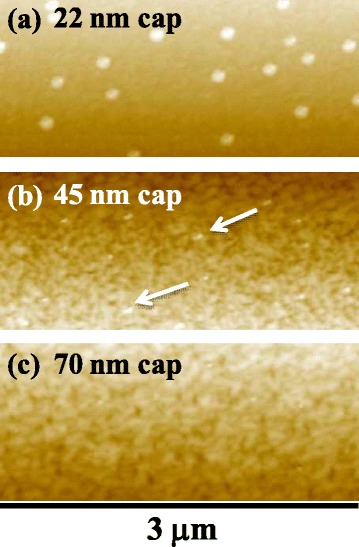
**AFM images (3 μm × 3 μm) of a surface. (a)** 22-nm, **(b)** 45-nm, and **(c)** 70-nm-thick Al_0.3_Ga_0.7_As capping for low-density GaAs DE QDs.

For low-density GaAs DE QDs, as shown in Figure 1c,d, the surface of Al_0.3_Ga_0.7_As-capped GaAs DE QDs was investigated by AFM. Figure 2 indicates AFM images of low-density GaAs DE QDs, with a 22, 45, and 70-nm capping. At 22- and 45-nm capping, the position of low-density GaAs DE QDs was observed. Flat and uniform AFM surfaces were found with the Al_0.3_Ga_0.7_As capping thickness of 70 and 105 nm (not shown here).

**Figure 3:**
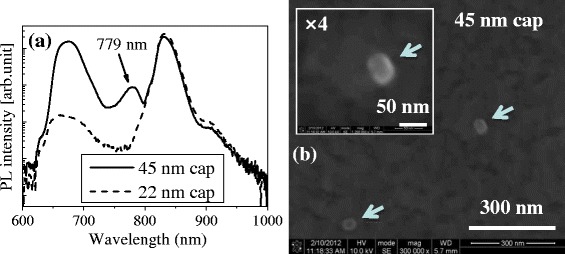
**Macro-PL and SEM image. (a)** Macro-PL (log scale) of low-density GaAs DE QDs with 22- and 45-nm cap at low temperature (12 K). The PL intensity was normalized with PL peak intensity of GaAs-related peak band around 830 nm. **(b)** SEM image (x300,000) of low-density GaAs DE QDs with 45-nm AlGaAs capping layer. The inset of Figure 3b is magnified by four for one QD.

For low-density GaAs DE QDs with 22- and 45-nm capping, a macro-PL at a low temperature (approximately 12 K) was measured, to verify an emission of low-density GaAs DE QDs, as shown in Figure 3a. Low-density GaAs DE QDs with a 45-nm-thick capping layer showed an emission at approximately 779 nm. However, 22-nm-capped GaAs DE QDs had no emission peak of QDs. An emission at approximately 779 nm for low-density GaAs DE QDs with a 45-nm capping matches the macro-PL result of 773-nm emission in Figure 1c. The PL bands around 660 and 830 nm shown in Figures 1c and 3a are attributed to Al_0.3_Ga_0.7_As and GaAs, respectively. The larger intensity of the PL band near 660 nm is found with the thicker AlGaAs capping layer in Figure 3a.

Figure 3b shows a SEM image of low-density GaAs DE QDs with a 45-nm cap, and arrows point out the position of GaAs DE QDs. Since SEM is widely used for a post-fabricating process, such as electron-beam lithography to form special structures like mesa or micro-pillar, it is significant that the position of QDs is observed on a SEM image. Furthermore, the wavelength of 779 nm is suitable for picking up appropriate single QDs in conventional cathode-luminescence apparatus, usually connected to modern SEM. The noteworthy result is that the density of GaAs DE QDs with a 45-nm-thick Al_0.3_Ga_0.7_As capping on the SEM image of Figure 3b corresponds to the density of Ga DE QDs (approximately 4 QDs/μm^2^) on the AFM image of Figure 1b. No change of density of QDs from droplets is due to i) immediate injection of As_4_ at near room temperature after Ga droplet growth and ii) suppression of Ostwald ripening considerably depending on growth temperature and growth interruption time [22]. This result mentions that prediction of the density of GaAs DE QDs at the step of Ga droplet formation is reliable, even after capping.

Here, it should be pointed out that the author cannot expect the optical properties of GaAs DE QDs with thin AlGaAs capping layer (45 nm) are the identical to that of GaAs DE QDs with 105-nm-thick AlGaAs capping layers. There will be the increasing effect of the surface as the thickness of AlGaAs decreases. The authors argued that the confinement of carrier by GaAs DE QDs was found as thin as 45 nm of AlGaAs capping by macro-PL, which was useful to plasmatic coupling application and can be locatable in SEM. In the near future, the authors will focus on the effect of thinness of capping on the individual QDs with μ-PL.

## Conclusions

In conclusion, 45-nm Al_0.3_Ga_0.7_As-covered GaAs DE QDs (approximately 4 QDs/μm^2^) have been retrievable on a SEM and an AFM. Low-density GaAs DE QDs with a 45-nm-thick capping layer showed the emission of a macro-PL at approximately 779 nm. For fully capped GaAs DE QDs (105-nm Al_0.3_Ga_0.7_As cap), a macro-PL shows an emission peak at approximately 773 nm, and an exciton/biexciton peak was verified at approximately 1.656 and approximately 1.655 eV, respectively, by μ-PL. Observation of these low-density GaAs DE QDs, that is, retrieving of an optically active single QD on the capped surface, would be of considerable impact to the mass production of devices using a single QD.
